# Treatment of dextran sodium sulfate-induced experimental colitis by adoptive transfer of peritoneal cells

**DOI:** 10.1038/srep16760

**Published:** 2015-11-13

**Authors:** Ting Liu, Jun Ren, Wei Wang, Xia-wei Wei, Guo-bo Shen, Yan-tong Liu, Min Luo, Guang-chao Xu, Bin Shao, Sen-yi Deng, Zhi-yao He, Xiao Liang, Yu Liu, Yan-Zhu Wen, Rong Xiang, Li Yang, Hong-xin Deng, Yu-quan Wei

**Affiliations:** 1 State Key Laboratory of Biotherapy and Cancer Center/National Collaborative Innovation Center for Biotherapy, West China Hospital, Sichuan University, Chengdu, Sichuan 610041, PR China; 2 Department of Immunology, Medical School of Nankai University, 94 Weijin Road, Tianjin 300071, PR China

## Abstract

The adoptive transfer of the natural regulatory B cells and macrophages should be a useful treatment for inflammation and autoimmune disease. However, it is usually difficult to isolate these cells from the tissues and expand them. Here, we investigated the feasibility of adoptively transferring peritoneal cells (PCs) as a treatment for DSS-induced colitis. We found that peritoneal cavity can provide an easily accessible site for harvesting enough number of PCs, namely, two-dose PCs for the treatment from a mouse in one operation. Adoptive therapy of these cells from healthy mice or those with disease is effectively in reducing the disease activity score. The natural B cells and macrophages of the infused PCs can selectively migrate to lesion sites and regulate the expression of Stat3, NF−κB, Smad3 and Smad7. Additionally, PCs exert dual activity of IL-10 and TGF-β secreted spontaneously by both peritoneal B cells and macrophages, which in turn enhance the induction of regulatory B cells and Macrophages in microenvironment of inflammation. Moreover, PCs can re-establish immunological tolerance in the OVA-immunized mice. Thus, our findings provide a new strategy for colitis therapy and could be of importance in additional exploration of other inflammation and autoimmune diseases therapy.

Inflammatory bowel diseases (IBD), including Crohn’s disease (CD) and ulcerative colitis (UC), are chronic inflammatory disorders of the gastrointestinal tract with high morbidity. Both CD and UC are characterized by weight loss, diarrhea and hematochezia^1^. The etiology and pathogenesis of IBD have been associated with many factors, including the interaction of environmental, genetic and immune factors, which can initiate an abnormal immune response and chronic inflammation^2^. Therefore, a cure for IBD would block inflammation and also re-establish immunological tolerance^3^. The current standard drug regimen focuses on suppression of inflammation using 5-aminosalicylic acid, corticosteroids and immunomodulators^4^,^5^. Although therapeutic efficacies have been enhanced by optimizing the standard drug regimen, adverse side effects and high relapse rates impede its further improvement. During the last decades, many new strategies have been developed to treat IBD. Infliximab, a monoclonal antibody to TNF-α, is broadly used, but this treatment may result in tuberculosis and chronic infection^6^,^7^. Cell therapy is a hot research topic in the treatment of IBD. It was reported that regulatory T (Treg) cells and mesenchymal stem cells (MSCs) could ameliorate murine autoimmune diseases^8^,^9^. However, further investigations of novel therapeutics are warranted.

The regulatory immune cells such as B cells and macrophages, etc. are widely distributed throughout the body, which can maintain immunological tolerance^10^,^11^. Regulatory B cells, a newly described subset of B cells, have been shown to negatively regulate autoimmunity and inflammation in numerous mouse models^11^. Previous studies have reported that wild type CD1d^hi^CD5^+^ splenic B cells significantly prolonged the survival of CD19 deficient-mice^12^. Phenotypically these B cells share surface markers with CD5^+^ B1a cells, which are characteristic by the production of high level of IL-10 and may be important in immune responses at mucosal surfaces^13^. Additionally, classically activated macrophages have long been known to play an important role in host defense. M2 macrophages are able to reduce the levels of inflammatory cytokines and secrete copious amounts of anti-inflammatory molecules like IL-10 and TGF-β, thereby down-regulating inflammatory processes in chronic inflammatory diseases. It is conceivable that the adoptive transfer of natural regulatory B cells and M2 macrophages could be an ideal treatment for IBD and other autoimmune diseases. However, the major limitation of this subset is that it is difficult to acquired sufficient number of regulatory B cells and M2 macrophages from tissues under non-elicited condition. Clinically, the IL-10-producing B cell subset characterized in humans normally represents 1% to 3% of spleen B cells and <1% of peripheral blood B cells^14^, and it is not feasible to isolate cells from patient’s spleen. Once isolated, they need to be expanded without the loss of their regulatory properties. However, the expansion of immune cells is potentially associated with serious adverse effects such as uncontrolled cell proliferation, pan immunosuppression and consequent tumor development^15^. Yet, it is still difficult to generate sufficient cell numbers of regulatory B cells and M2 macrophages for adoptive therapy by expansion. Therefore, further investigations of simple, rapid and safe therapeutics by the use of natural regulatory B cells and macrophages are warranted.

The peritoneal cavity is a unique compartment within which a variety of immune cells reside. Peritoneal cells (PCs) in the peritoneal cavity are composed of 50–60% B cells, ~30% macrophages and 5–10% T cells^16^. Recently studies have shown that the majority of B1a cells are located in peritoneal and pleural cavities with a high CD5 and CD11b phenotype^17^. Additionally, PCs strongly expressed CD206 mRNA, which is characteristic of M2 macrophages^18^. Clinically, abdominal paracentesis is a fairly simple procedure, in which the peritoneal cavity is punctured by a needle to sample peritoneal fluid. The procedure generally is safe, rapid and does not require sedation. Therefore, the peritoneal cavity provides an easily accessible site for harvesting a large number of non-manipulated Peritoneal cells (PCs), including B1a cells and M2 macrophages.

It is conceivable that the adoptive transfer of freshly collected PCs without any additional *ex vivo* culture should be used as a new approach for inflammation and autoimmune disease therapy. Here, we mainly explored whether adoptive transfer of PCs alone might represent a novel method to mitigate inflammation in the DSS-induced colitis model. We also investigated the PC cell type that alleviates the severity of the colitis. In addition, the underlying mechanisms by which PCs function to treat DSS-induced colitis were examined. To our knowledge, prior to the presented study here, no study has reported therapeutic effects initiated by the transfer of PCs in inflammation and autoimmune disease.

## Results

### Adoptive transfer of PCs treated DSS-induced colitis

**Figure 1:**
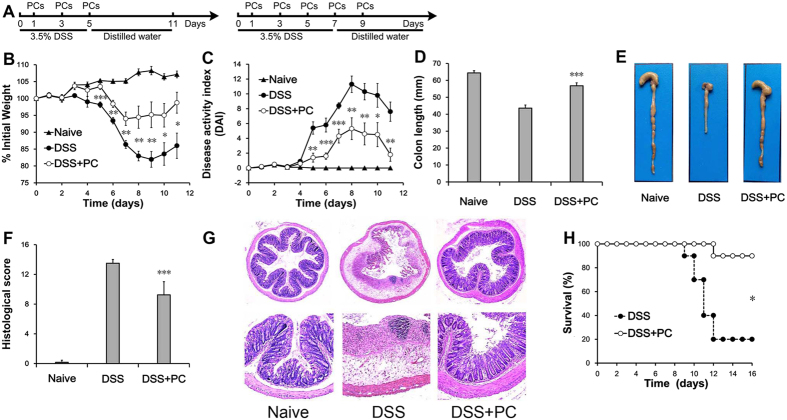
Adoptive transfer of PCs treated DSS-induced colitis. (**A**) Colitis was induced by the oral administration of 3.5% DSS for 5 days. The DSS and DSS + PC groups were intravenously injected with 100 μl saline or 1 × 10^6^ PCs, respectively, on days 1, 3 and 5. Mice that were orally administered normal distilled water were used as controls. The time courses of (**B**) body weight change and (**C**) disease activity index (DAI) were monitored daily. On day 11, (**D,E**) colon length and (**F,G**) colon histopathology were analyzed. (**H**) Mice from the DSS and DSS + PC groups were further subjected to DSS until day 7, when they were returned to normal distilled water and assessed for survival. These observations clearly demonstrate the therapeutic potential of peritoneal cells to reduce the development and progression of inflammatory changes caused by DSS toxicity. Data presented indicate the mean ± SEM of ten mice. Similar results were observed in three independent experiments. *p < 0.05, **p < 0.01 and ***p < 0.001 (Naive and DSS + PC versus DSS, ANOVA test, and Wilcoxon Mann–Whitney test).

DSS-induced colitis is a classical disease model that results in the stable occurrence of disease and many similarities with the clinical manifestations of colitis in human IBDs. We measured the optimal concentration of PCs by several preliminary experiments, and then treated the mice on days 1, 3, and 5 with optimal 1 × 10^6^ freshly isolated PCs. The transfer of peritoneal cells was effective in inhibiting weight loss during DSS-induced colitis. In the DSS group, mice weighed 81.9 ± 2.3% of the original bodyweight at Day 9 (Fig. 1B). Mice in the DSS + PC group weighed 95.2 ± 3.8% of the original bodyweight, respectively. After removal of DSS from the drinking water, mice in the DSS group continued to lose weight, whereas the body weight of DSS + PC treated mice recovered.

The disease activity index (DAI) is clinically used to describe the severity of colitis. The DAI of DSS + PC treated mice was lower than that of the DSS group mice (Fig. 1C). DSS-treated mice gradually displayed changes in bodyweight and stool consistency. Loose stool with the presence of blood were clearly detected at day 6 and diarrhea with blood and gross bleeding were observed at day 7. On day 10, 80% of DSS treated mice were found to show gross bleeding. By contrast, the DSS + PC treated mice displayed less severe and delayed colitis symptoms.

To macroscopically assess inflammation, we carefully inspected changes in the gross appearance of colons. Compared to the DSS mice, mice that received PCs showed less severe inflammation (because the shortened colon lengths of the DSS + PC mice were lower than DSS group (11.8% versus 32.3%, p < 0.001, Fig. 1D,E).

Additional histological analysis supported the macroscopic assessments. The DSS-treated mice exhibited extensively distributed inflammation with loss of the entire crypts and epithelial surface, while the DSS + PC treated mice had mild or moderate inflammation (Fig. 1F). The histopathological colitis score in the DSS + PC-treated mice was lower than the score of the DSS-treated mice (p < 0.001, Fig. 1G).

Additionally, the survival rate of DSS + PC-treated mice was significantly prolonged compared to DSS-treated mice (Fig. 1H). All DSS-treated mice died between days 9 and 12, whereas the DSS + PC-treated mice died on day 12. Furthermore, 20% of the DSS-treated mice survived, whereas 90% of the DSS + PC-treated mice survived and achieved complete restoration of original body weight (p < 0.05).

These observations clearly demonstrate the ability of peritoneal cells to reduce the development and progression of inflammatory changes caused by DSS toxicity and enhanced the process of mucosal healing and recovery.

### Adoptive transfer of PCs reduced pro-inflammatory cytokine and induced immunosuppressive cytokines on both gene and protein levels

**Figure 2:**
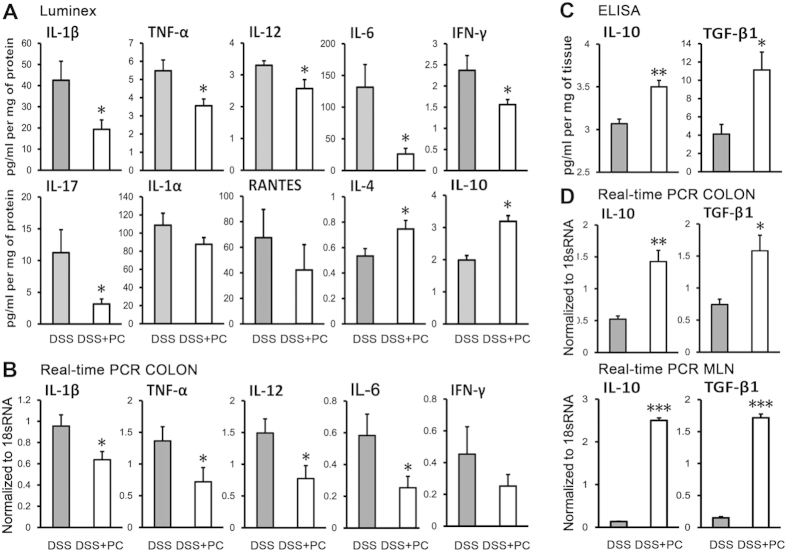
PCs treatment alters the cytokine profile in colitic mice. On day11, colon tissues and mesenteric lymph nodes (MLN) were harvested. (**A**) The protein levels of cytokines in colon tissues (COLON) were measured by luminex (n = 8–10/group). (**C**) IL-10 and TGF-β1 were further measured by ELISA (n = 4–5/group). The expression of the indicated genes in (**B,D**) colon tissues and mesenteric lymph nodes (MLN) were measured by real-time PCR normalized to 18sRNA (n = 3–5/group). These observations demonstrate that the reduction of pro-inflammatory cytokine levels and induction of immunosuppressive cytokines on both gene and protein levels can be ascribed to the remission of disease. Data presented indicate the mean normalized value ± SEM. Similar results were observed in three independent experiments. *p < 0.05, **p < 0.01 and ***p < 0.001 (DSS + PC versus DSS, Student’s t-test).

We investigated whether PC treatment could alter the levels and expression of pro-inflammatory and anti-inflammatory cytokines in colon tissues (COLON) and mesenteric lymph nodes (MLN). The protein levels of the pro-inflammatory cytokines IL-1β, TNF-α, IL-12, IL-6, IFN-γ, IL-17 and IL-1α were decreased in mice that received PCs, while the immunosuppressive cytokines IL-4, IL-10 and TGF-β1 were increased (Fig. 2A,C). Furthermore, we measured the mRNA levels of IL-1β, TNF-α, IL-12, IL-6, IFN-γ, IL-10 and TGF-β1, and got consistent results (Fig. 2B,D). Notably, the IL-10 and TGF-β1 in mesenteric lymph nodes were increased approximately 10-fold in mice that received PCs (Fig. 2D). These findings indicate the decreased pro-inflammatory cytokines and increased immunosuppressive cytokines on both gene and protein levels, caused by PCs-transfer in mice with DSS-induced colitis.

### Adoptive transfer of peritoneal B cells or macrophages alone can treat DSS-induced colitis

**Figure 3:**
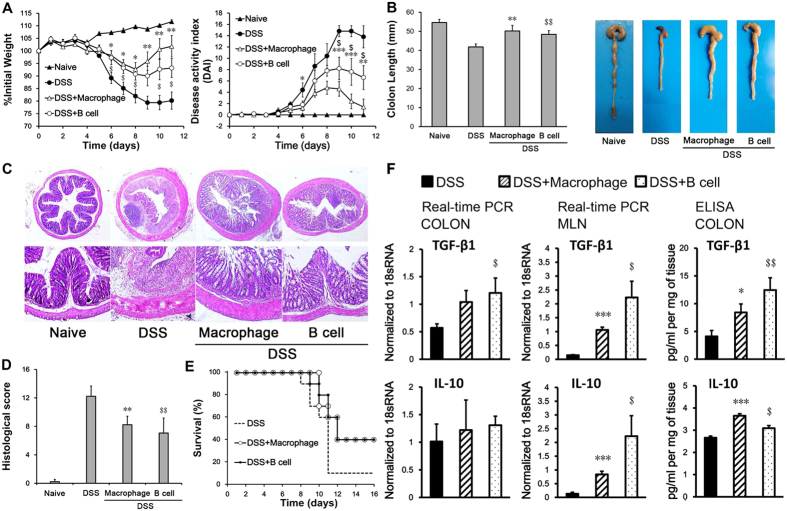
Adoptive transfer of peritoneal B cells or macrophages alone can ameliorate DSS-induced colitis. Colitis was induced by oral administration of 3.5% DSS for 5 days. The DSS, DSS + B cell and DSS + Macrophage groups were intravenously injected with 100 μl saline, 1 × 10^6^ peritoneal B cells or 1 × 10^6^ peritoneal macrophages, respectively, on days 1, 3 and 5. Mice that were orally administered normal distilled water were used as controls. The time courses of (**A**) body weight change and disease activity index (DAI) were monitored daily. On day 11, (**B**) colon length and (**C**) Histopathology of the colon and (**D**) representative colon sections (H&E stained) were analyzed. Data presented indicate the mean ± SEM of ten mice. (**E**) Mice from the DSS, DSS + B cell and DSS + Macrophage groups were further subjected to DSS until day 7, when they were returned to normal distilled water and assessed for survival. Data presented indicate the mean ± SEM of ten mice. (**F**) Colon tissue (COLON) and mesenteric lymph nodes (MLN) were harvested to measure the expression of the IL-10 and TGF-β1 by real-time PCR and ELISA. Data presented indicate the mean normalized value ± SEM (n = 4–5/group).These observations clearly demonstrated that the peritoneal B cells and macrophages are both competent DSS-induced colitis cell therapies, which can ameliorate disease in a remarkably high percentage of recipient hosts. Similar results were observed in three independent experiments *p < 0.05, **p < 0.01 and ***p < 0.001, DSS + Macrophage versus DSS; ^$^p < 0.05, ^$$^p < 0.01 and ^$$$^p < 0.001, DSS + B cell versus DSS (ANOVA test, and Wilcoxon Mann–Whitney test).

To determine the cell type by which PCs attenuate experimentally induced colitis, we separated macrophages and B cells from total PCs, detected the purity by flow cytometry and intravenously administered them into DSS-treated mice. Interestingly, mice that received either peritoneal macrophages or B cells exhibited attenuated severity of colitis. Similar to the mice that received total PCs, mice administered peritoneal macrophages or B cells had an increased rate of bodyweight recovery and a lower DAI score (Fig. 3A). Compared with the DSS-treated group, mice that received peritoneal macrophages or B cells had longer colon lengths (Fig. 3B) and exhibited improved mucosal recovery and reduced inflammation (Fig. 3C,D). The survival rate of the peritoneal B cell or macrophage recipient mice was higher than that of the DSS group, although it remained lower than that of the total PC-treated group (Fig. 3E and 1H). Similarly, TGF-β1 and IL-10 in the MLN and COLON were also increased in peritoneal macrophage- and B cell-treated mice (Fig. 3F).

These observations suggest that peritoneal macrophages and B cells are responsible for the therapeutic effects of PCs in colitis, as peritoneal macrophages and B cells both have the ability to reduce the development and progression of colitis induced by DSS.

### Both IL-10 and TGF-β are key mediators of PC-dependent immunosuppression

The intravenous transfer of PCs or PC-derived cells altered the expression of cytokines closely linked to inflammation, both in COLON and MLN (Figs 2 and 3). Among these cytokines, levels of IL-10 and TGF-β, two cytokines pivotal for immunosuppression, were increased. Thus, we inferred that IL-10 and TGF-β play key roles in PC-mediated immunosuppression. To test whether cytokines produced by PCs could affect the anti-colitic efficacy of PCs, we test the ability of PCs to produce immunosuppressive cytokines, examined the anti-colitic efficacy of PCs from cytokine knock-out mice and also systemically neutralized both IL-10 and TGF-β using specific antibodies.

**Figure 4:**
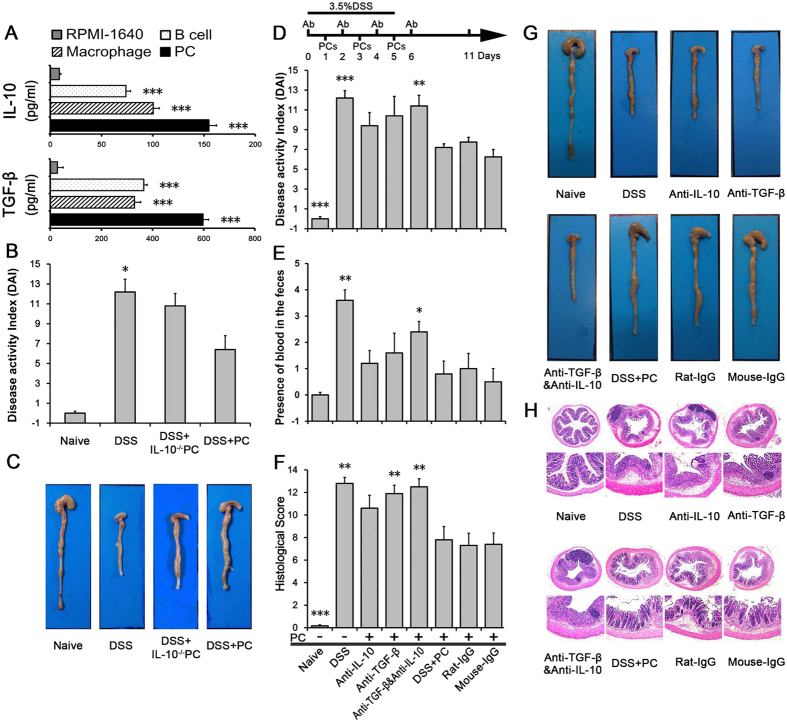
IL-10 and TGF-β are key mediators by which PCs execute immunosuppression. (**A**) PCs, peritoneal B cells and macrophages isolated from C57BL/6 mice were cultured *in vitro*. The levels of IL-10 and TGF-β1 in culture supernatants were measured by ELISA. PCs from IL-10^−/−^ mice were intravenously injected for therapy. On day9, the (**B**) disease activity index and (**C**) colon length were analyzed. These findings suggest that PCs can produce immunosuppression cytokines and IL-10 play a partial role in the treatment of colitis. Furthermore, (**D**) Anti-IL-10 Ab (total, 250 μg/mouse) and/or anti-TGF-β-1, -2, -3 Ab (total, 250 μg/mouse) were intraperitoneally administered 1 day before and 1 day after PC treatment, which reversed the anti-colitic effects of PCs as assessed by disease activity index, the scores of presence of blood in the feces, (**E**) colon length and (**F**) histopathology score. In time-matched rat-IgG and mouse-IgG control mice, DSS elicited colitis and could be suppressed by PC injection. These observations indicate the essential role of IL-10 and TGF-β in the anti-inflammatory effects mediated by PCs. Data presented indicates the mean ± SEM of five mice. *p < 0.05, **p < 0.01 and ***p < 0.001 (B cell, Macrophage and PC versus RPMI-1640; Naive, DSS, DSS + IL-10^−/−^, Anti-IL-10, Anti-TGF-β, Anti-TGF-β&IL-10, Rat-IgG and Mouse-IgG versus DSS + PC, ANOVA test).

We found that total PCs, peritoneal macrophages and B cells were able to produce IL-10 and TGF-β1 *in vitro* (Fig. 4A). Also, peritoneal macrophages produced more IL-10 and less TGF-β1 when compared to peritoneal B cells. Next, we administered PCs from IL-10 knock-out mice (IL-10^−/−^ PCs) to examine the effect of IL-10 on the anti-colitic efficacy of PCs. IL-10^−/−^ PCs remained able to produce TGF-β and ameliorate DSS-induced colitis, but were less efficacious than PCs from wild-type mice, as indicated by the disease activity score and colon length (Fig. 4B,C). These findings suggest that IL-10 and TGF-β play a partial role in the treatment of inflammatory bowel disease. Furthermore, neutralizing IL-10 and TGF-β significantly attenuated the therapeutic efficacy of transferred PCs. The average score of the presence of blood in the faces was higher, colon length was shorter and colon inflammation was more severe in IL-10- or TGF-β-neutralized mice than in the PC-treated group (Fig. 4D–H). As indicated by DAI scores, neutralizing antibody-treated mice had higher scores, especially mice that received both IL-10 and TGF-β neutralizing antibodies (Fig. 4D).

These observations suggest that the dual activity of IL-10 and TGF-β secreted spontaneously by natural regulatory B cells or macrophages of the infused PCs are responsible for the therapeutic effect of PCs in colitis.

### PCs can selectively migrate to the specific sites of inflammation and regulate the expression of Stat3, NF-κB and Smad3

**Figure 5:**
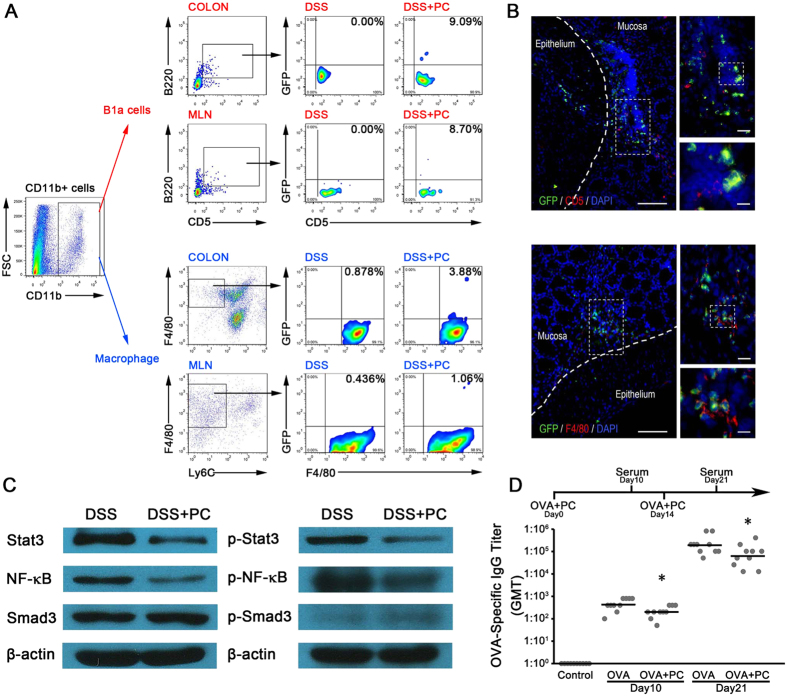
PCs can selectively migrate to the specific sites of inflammation and regulate the immune response. (**A**) GFP^+^-PCs were intravenously injected into the colitis mice, the expression of GFP^+^-macro-phages and -B cells in COLON and MLN pooled from 5 mice are measured by FACS. (**B**) Immunofluorescence of DAPI-stained GFP + F4/80 + macrophages and GFP + CD5 + B cells can migrate to the specific sites of inflammation**. (C)** The expressions of Stat3, NF-κB and Smad3 in colon tissue of colitis mice treated with PCs or not, were measured by western blot. Each replicate represents data obtained from 5 pooled colons. (**F**) Serum titers of OVA-specific IgG were measured on indicated days in mice immunized subcutaneously with OVA alone, or with OVA and peritoneal cells (n = 10/group). These observations indicate that the natural B cells and macrophages of the infused PCs can selectively migrate to lesion sites, block inflammation and also re-establish immunological tolerance. The experiments were performed at least three times, yielding similar results. *p < 0.05, **p < 0.01 and ***p < 0.001 (OVA + PC versus OVA, Student’s t-test).

To investigate whether the adoptive transferred PCs could selectively migrate to the specific sites of inflammation, PCs from Green-fluorescent protein (GFP)-transgenic mice of C57BL/6 background were used to trace the peritoneal cells *in vivo*. Colitic mice that received GFP + PCs showed more GFP^+^ Macrophages (3.88% versus 0.878% in COLON, 1.06% versus 0.436% in MLN) and GFP^+^ B1a cells (9.09% versus 0.00% in COLON, 8.70% versus 0.00% in MLN) (Fig. 5A). In immunofluorescence test, we found that the GFP^+^-PCs can selectively migrate to the specific sites of inflammation (Fig. 5B).

Recent works suggest that development of colonic inflammation is associated with the induction of Stat3, Smad3 and Nuclear factor-κB (NF-κB) activity in intestinal epithelial cells, and the levels of phosphorylation correlated to the severity of colitis^19^,^20^,^21^,^22^,^23^,^24^. In addition, Stat3 and Smad3 is downstream signaling molecules of the IL-10 and TGF-β receptor, respectively^19^,^21^. Therefore, we hypothesis that IL-10 and TGF-β elicit protective effects through the reduction of Stat3, Smad3 and NF-κB. Indeed, we found in mice that the reduction of Stat3, Smad3 and NF-κB contribute to anti-colitic efficacy of PCs (Fig. 5C). Although, total Smad3 was equally expressed in DSS and DSS + PC group, the phosphorylation of Smad3 was increased in DSS + PC group.

### PCs reduced OVA specific antibody and re-establish immunological tolerance

IL-10 and TGF-β are two well-defined immunosuppressive cytokines that can comprehensively inhibit immune responses. Total PCs, peritoneal macrophages and B cells were able to produce IL-10 and TGF-β1. We therefore inferred that the adoptive transfer of PCs might elicit comprehensive immunosuppression. To test our hypothesis, we measured antibody titers after PC administration in OVA-immunized mice. On days 0 and 14, mice were subcutaneously immunized with OVA. At the same time, PCs were administered intravenously. OVA-specific antibody titers were measured in serum collected on days 10 and 21 (Fig. 5D). The adoptive transfer of PCs reduced the titers of OVA-specific antibodies in the serum, suggesting the comprehensive inhibition of immune response by PCs transfer. Therefore, the extent of PC adoptive cell therapy could be broadened to other autoimmune diseases.

### PCs activated TGF-β/Smad pathway and induced regulatory cells in inflammation sites

**Figure 6:**
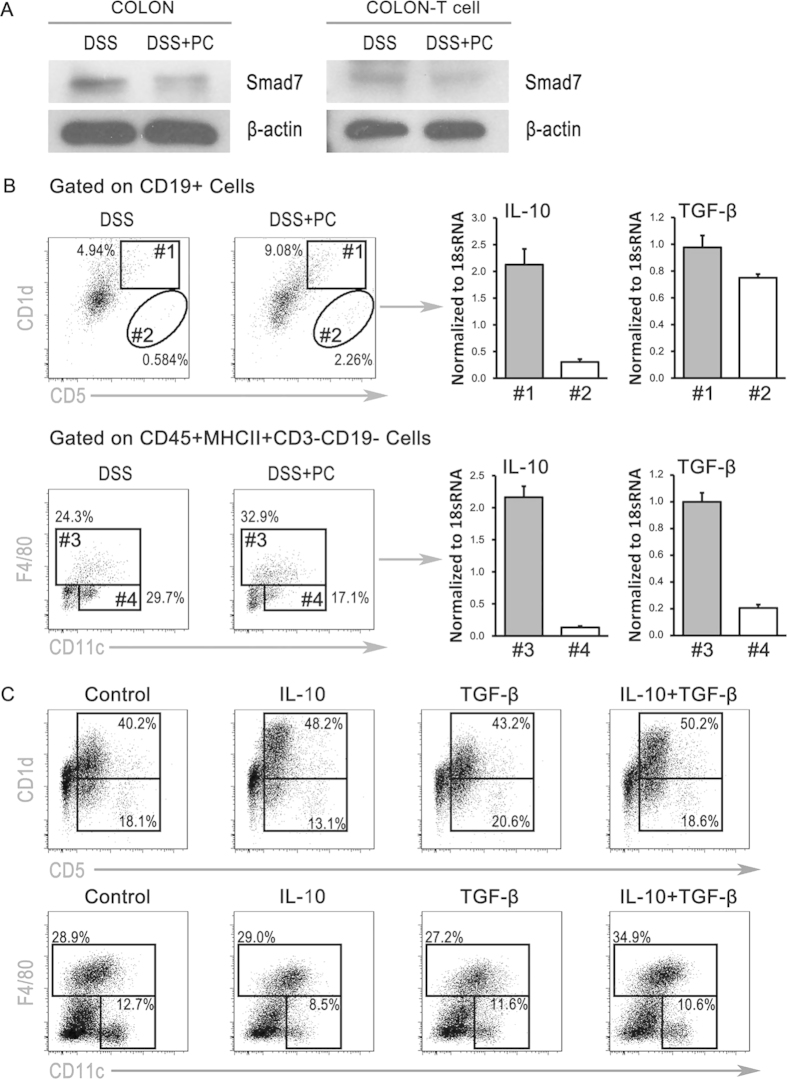
PCs inhibited smad7 and induced regulatory macrophages and B cells in colon. (**A**) The expression of Smad7 in the mucosal and mucosal T cells of colitis mice treated with PCs or not, was measured by western blot. (**B**) Representative FACS analysis showing the gating strategy to define colonic anti-inflammatory B cells and macrophages in colon tissue, and the specific cell sorting was performed by BD FACS Aria II. B cells were firstly gated on CD19 + cells and subdivided into CD1d + CD5 + B cells and CD1d-CD5 + B cells. Macrophages were gated on CD45 + MHCII + CD3-CD19- cells, and then subdivided into anti-inflammatory macrophages and inflammatory DCs based on F4/80 and CD11c. The mRNA levels of IL-10 and TGF-β1 in CD1d + CD5 + B cells, CD1d-CD5 + B cells, anti-inflammation macrophages and inflammatory DCs were measured by RT-PCR. (**C**) The lymphocytes and other mononuclear cells isolated from BM were cultured with IL-10/TGF-β1 or not. After 24 hours, the cells were collected and measured by FACS. Each replicate represents data obtained from 5–10 pooled colons. The experiments were performed at least three times, yielding similar results.

Smad7 is an inhibitor of TGF-β signaling, which is over-expression in the mucosa and mucosal T cells of patients with inflammatory bowel disease^25^,^26^. In this study, PCs can reduce the expression of Smad7 in colon, especially in mucosal T cells (Fig. 6A). Additionally, PCs can induce the regulatory macrophage and regulatory B cell in the colon of colitic mice. Both induced regulatory macrophage and B cell were strongly expressing IL-10 and TGF-β1 mRNA (Fig. 6B). We cultured the BM cells with recombinant TGF-β1 and IL-10 *in vitro*, and found that both cytokines were responsible for the generation of regulatory cells (Fig. 6C).

### PCs from colitic mice retain the ability to attenuate the severity of colitis

**Figure 7:**
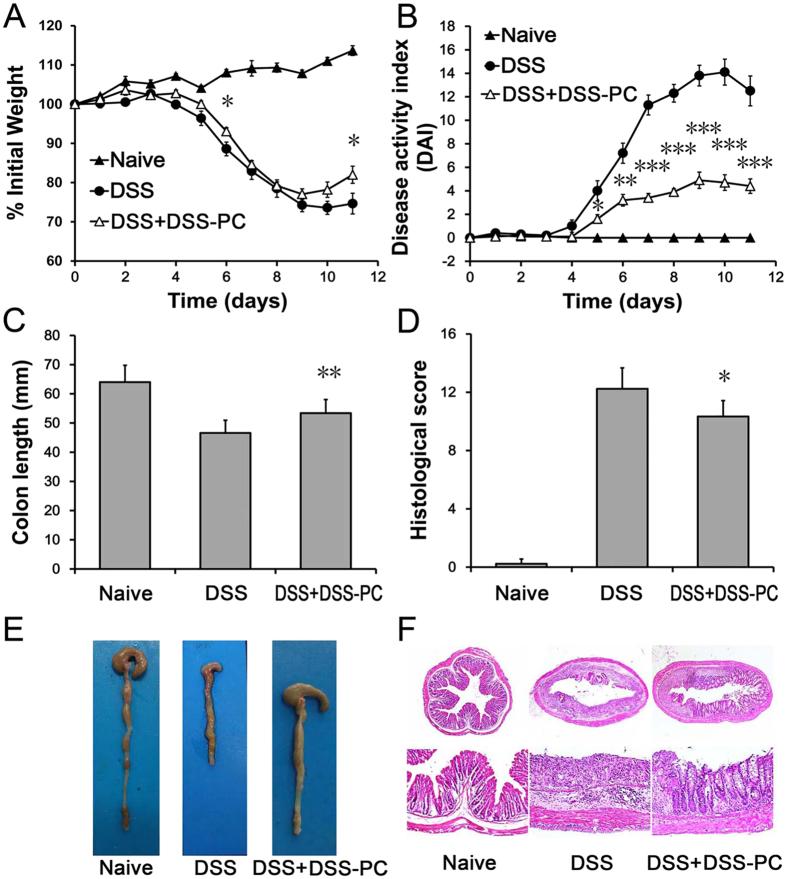
Transferred PCs from colitic mice retain the ability to attenuate the severity of colitis. 1 × 10^6^ PCs from colitic mice were intravenously injected on days 1, 3 and 5 for therapy. The time course of (**A**) body weight change and (**B**) disease activity index (DAI) were monitored daily. On day 11, (**C,E**) colon length, (**D**) colon histopathology and **(F)** representative colon sections (H&E stained) were analyzed. These observations indicate that transferred PCs from colitic mice could mitigate the severity of colitis, which suggests the clinical availability of PCs from patients themselves. Data presented indicate the mean ± SEM of ten mice. The experiments were performed at least twice, yielding similar results. *p < 0.05, **p < 0.01 and ***p < 0.001 (DSS + DSS-PC versus DSS, Student’s t-test).

Although PCs could effectively treat colitis, there are many obstacles for the use of PCs in the clinic. Most notably, it is difficult to find suitable donors to provide MHC-matched PCs. Ideal PCs would be harvested from the patients themselves. To determine whether PCs from colitic mice could effectively treat colitis, we intravenously administered PCs from DSS-induced colitic mice to treat colitis. Intriguingly, PCs from colitic mice remained capable of inhibiting the inflammation in DSS-induced mice, as indicated by bodyweight recovery, DAI score, colon length and colon inflammation (Fig. 7A–F). These findings suggested the clinical availability of PCs from patients themselves.

## Discussion

Several observations have been made in this study concerning the PCs, natural regulatory B cells and macrophages, and their adoptive therapy for DSS-induced colitis. In the present study, adoptive therapy of freshly isolated PCs from healthy mice or those with disease is effective in the treatment of DSS-induced colitis. The dual activity of IL-10 and TGF-β secreted spontaneously by natural regulatory B cells or macrophages of the infused PCs are probably responsible for the therapeutic effect. These suggestions are supported by our finding in this study. Adoptive transfer of peritoneal B cells or macrophages alone isolated from PCs is also effective in the treatment. The B cells or macrophages of the infused PCs migrated to the lesion sites, induced the regulatory cells and regulate the expression of Stat3, NF-κB, Smad3 and Smad7. Adoptive transfer of PCs, B cells or macrophages reduced pro-inflammatory cytokine levels and induced immunosuppressive cytokines. Both natural regulatory B cell and macrophage isolated from PCs were able to spontaneously produce IL-10 and TGF-β1. Neutralizing IL-10 and TGF-β by the antibodies against IL-10 and TGF-β significantly attenuated the therapeutic efficacy of transferred PCs.

It is known that both IL-10 and TGF-β have an important role in the establishment of tolerance and suppression of inflammation diseases^27^. Interleukin-10 (IL-10) is a pluripotent cytokine that is produced by different cell types including B lymphocytes and macrophages^28^. As an anti-inflammatory cytokine, IL-10 has the ability to differentially affect the function of different subsets of immune cells, affecting both the innate immune system and the adaptive immune system^29^. The link between IL-10 and regulation of colonic immune responses was suggested by the finding that mice and humans deficient in either IL-10 or IL-10 receptor are highly susceptible to autoimmune disease and spontaneously develop intestinal inflammation^30^,^31^. It is interesting to note that the injection of mice with recombinant IL-10 can inhibit chronic inflammatory diseases such as Crohn’s disease^32^,^33^. Thus, IL-10 has a potent immunosuppressive capacity that has an essential role not only in the establishment of tolerance to allergens, but also in protecting the host from exaggerated inflammatory responses to pathogens as well as autoimmune disease^34^. Transforming growth factor β (TGF-β) is another regulatory cytokines with pleiotropic functions, and it is also produced by different cell types including B lymphocytes and macrophages^34^. As an anti-inflammatory cytokine, TGF-β has the ability to modulate immune responses, especially in control of tolerance to self-antigens and T cell differentiation^35^. In the gut, TGF-β is an important epithelial cell growth factor and is a potent inducer of intestinal epithelium repair after mucosal injury^36^. However, the administration of recombinant TGF-β intraperitoneally is ineffective at treating experimental colitis, because smad7 is over-expression in the mucosa and mucosal T cells of patients with inflammatory bowel disease^25^,^26^. It is reported that the expression of Smad7 is induced by IFN-γ as a negative regulator of the TGF-β/Smad pathway^37^. Therefore, inhibition of IFN-γ and smad7 can restored TGF-β signaling. These studies mentioned above supported our suggestion that both IL-10 and TGF-β secreted spontaneously by natural regulatory B cells or macrophages of the infused PCs are probably responsible for the therapeutic effect. In contrast to the therapy based on IL-10 or TGF-β alone, the adoptive transferred PCs may exert dual therapeutic activity of IL-10 and TGF-β from both natural regulatory B cells and macrophages. This conclusion is drawn from the following experimental data in the present study: (a) The adoptive transfer of peritoneal B cells or macrophages alone is less effective than total PCs; (b) The anti-inflammatory ability of PCs from IL-10^−/−^ mice was lower than PCs from wild-type mice; (c) Either blockade of IL-10 or TGF-β, exert a partial role in the therapeutic effect. (d) PCs inhibited the smad7 in mucosal T cells and restored TGF-β signaling.

We found in the present study that intravenously administered natural B cells and macrophages of the infused PCs can selectively migrate to lesion sites. Thus, PCs have a potential target property that can accumulate to the specific sites of inflammation and regulate the expression of Stat3, NF-κB, Smad3 and Smad7 in colon. These results are highly consistent with recent report that development of colonic inflammation is associated with the induction of Stat3 and NF-κB activity in intestinal epithelial cells, and the levels of phosphorylation correlated to the severity of colitis^20^,^23^. Animals with disruption of smad3 exhibit abnormal immune function and develop inflammation in intestine^21^. In addition, Stat3 and Smad3 are the downstream signaling molecules of the IL-10 and TGF-β receptor, respectively^21^,^38^. Additionally, PCs can switch the differentiation of monocytes from inflammatory dendritic cells to anti-inflammatory macrophages, suppressing the differentiation of Th17^39^. PCs can also induce the CD5 + regulatory B cells in colon. In this study, both induced regulatory macrophage and B cell were strongly expressing IL-10 and TGF-β1 mRNA, which in turn enhance the function of peritoneal cells.

The main impediment that has hampered the use of natural B cells and macrophages therapy is that it is difficult to specifically isolate immunosuppressive cells and expand them for use in adoptive cell therapy clinically. PCs have many advantages when they are adoptively transferred for treating autoimmune diseases. First, PCs are highly bio-available. The peritoneal cavity provides an easily accessible site for harvesting a large number of non-manipulated PCs, namely, two-dose PCs for the treatment from a mouse in one operation. Notably, abdominal paracentesis is a fairly simple, safe and rapid procedure in clinical practice, in which the peritoneal cavity is punctured by a needle to sample peritoneal fluid. Second, PCs from colitic mice remained capable of inhibiting the inflammation in DSS-induced mice, suggesting that autologous PCs from patients themselves can be used in the treatment of autoimmunity. Additionally, freshly collected PCs can be immediately intravenously administered without any additional *ex vivo* culture. Therefore, the safety and ease of PC manipulation is enhanced. In our study, none of transplant rejection, pulmonary embolism and cytotoxicity is observed. Third, intravenously administered natural B cells and macrophages of the infused PCs can selectively migrate to lesion sites and regulate immune response. In the inflammation bowel disease, the endogenous PCs can migrate to the gut via the lymphatics and blood circulation, and are restricted almost exclusively to follicular structures present in the gut wall, such as Peyer patches and solitary intestinal lymphoid tissue, prior to migrating to the lesion sites. Indeed, few PCs enter into the circulation system and finally migrate to the specific inflammation sites, partially due to receptors in the peritoneal cavity specially recognizing the PCs, restricting the migration of PCs^40^,^41^. While, the isolated and intravenously infused PCs migrate to the lesion sites directly through blood circulation. In our test, the adoptive transfer of GFP labelled PCs can migrate to the specific sites of the inflammation within one hour (Fig. 5A,B). What’s more, the injury of DSS-induced colitis is starting from the mucosa to muscularis externa. Therefore, it is reasonable that the isolated and intravenously infused PCs are more efficiently recruited toward the lesion sites than endogenous PCs in the DSS-induced colitis mice model. Fourth, both natural regulatory B cell and macrophage isolated from PCs were able to spontaneously produce IL-10 and TGF-β1. Both IL-10 and TGF-β are probably responsible for the therapeutic effect of PCs. This multiple superposition effect enhanced the autoimmune therapeutic effect of PCs. Finally, the adoptive transfer of PCs reduced the titers of OVA-specific antibodies in the OVA-immunized mice. Therefore, the extent of PC adoptive cell therapy could be broadened to other autoimmune diseases.

Taken together, these observations may provide a new strategy for the adoptive therapy of the natural regulatory B cells and macrophages by the intravenously administered PCs for autoimmune diseases.

## Methods

### Mice

Female C57BL/6 6–7 weeks of age were purchased from Vital River, Peking, China. IL-10 knockout mice were purchased from the Jackson laboratory. Green-fluorescent protein (GFP)-transgenic mice were provided by the State Key Laboratory of Biotherapy and Cancer Center (Sichuan, China). All mice were housed and fed in a dedicated pathogen-free facility and maintained at 22 °C under 12-hour day and night cycles throughout the experiment. Animal experiments were performed according to the guideline of the Animal Care and Use Committee of Sichuan University (Chengdu, Sichuan, China) and approved by animal association.

### Study design

Female C57BL/6 mice were exposed to 3.5% dextran sodium sulfate polymers (DSS, MW 36–50 kDa, MP Biomedicals, OH, USA) in drinking water for 5 days, following by normal distilled water thereafter. On days 1, 3, and 5, isolated PCs, macrophages, or B cells were intravenously injected and the clinical disease severity was monitored daily.

The mice were randomly divided into seven groups. Mice in Naive group were orally administered with distilled water only. The DSS group was treated with DSS and intravenously injected with saline. The DSS + PC group was treated with DSS and intravenously injected with 1 × 10^6^ peritoneal cells. For the mechanistic studies, two groups were added. Mice in DSS + B cell and DSS + Macrophage group were treated with DSS and intravenously injected with 1 × 10^6^ B cells or macrophages, respectively. The DSS + DSS-PC group was treated with DSS and intravenously injected with 1 × 10^6^ peritoneal cells from DSS-induced colitic mice, which had been exposed to 3.5% DSS in drinking water for 5 days and distilled water for 2 days thereafter. Finally, in the DSS + IL-10^−/−^ PC group, DSS treated mice were intravenously injected with 1 × 10^6^ PCs from IL-10^−/−^ mice.

For the survival study, 10 mice each in the DSS, DSS + PC, DSS + B cell, and DSS + Macrophage groups were subjected to DSS until day 7, when they were returned to normal distilled water and monitored for survival^42^. The observation period lasted for 60 days.

### Cell isolation

Mice were injected intraperitoneally with 5–8 ml RPMI-1640 (Gibco, CA, USA). After abdominal kneading, peritoneal cells were harvested using a syringe^43^. Mouse peritoneal macrophages were obtained by culturing the PCs in RPMI-1640 containing 20% fetal bovine serum for 2 hours at 37 °C. After incubation, adherent cells were collected^44^,^45^. CD19^+^ B cells were separated from total PCs using a BD FACS Aria II cell sorter. All cells were resuspended in RPMI-1640 at a concentration of 1 × 10^6^ cells/100 μl and detected the purity by flow cytometry.

### Gross examination and histology

The clinical disease severity in each group was monitored daily, and scored according to the previous report^46^ and is described as follows: a) general appearance: normal = 0; piloerection = 1; lethargy and piloerection = 2; motionless, sickly = 4; b) weight loss: no change = 0; < 5% = 1; 6%–10% = 2; 11%–20% = 3; > 20% = 4; c) feces consistency: normal = 0; pasty, semiformed = 2; liquid, sticky, or unable to defecate after 5 minutes = 4; and d) rectal bleeding: no blood = 0; visible blood in rectum = 2; visible blood on fur = 4. For hematoxylin and eosin (H&E), the entire colon was removed and measured for length. Then, the colon was divided into five equal segments and fixed with 4% paraformaldehyde. The 3–4μm sectioned colon was stained with H&E and examined at 200 × magnification by light microscopy (Leica DFC425C, LAS V3.8 software). Histopathological evaluation was graded using a previously validated scoring system^47^: a) inflammation severity: none = 0; slight = 1; moderate = 2; severe = 3; b) depth of injury: none = 0; mucosal = 1; mucosal and submucosal = 2; transmural = 3; c) crypt damage: none = 0; basal one-third damaged = 1; basal two-thirds damaged = 2; only surface epithelium intact = 3; entire crypt and epithelium lost = 4; d) percentage of area involved: none = 0; 1%–25% = 1; 26%–50% = 2; 51%–75% = 3; 76%–100% = 4. The final scores are the averages of all individual scores of 10 pieces per colon.

### Cytokine measurements

Total RNA was extracted using a RNA extraction kit (Axygen, USA). Template cDNA was synthesized using the Primescript RT reagent kit with gDNA eraser (Takara, Tokyo, Japan). Real-time quantitative PCR primers were performed using the following primers: IL-1β-specific forward: 5′-TGTAATGAAAGACGGCACACC-3′,

IL-1β-reverse primer: 5′-TCTTCTTTGGGTATTGCTTGG-3′,

TNF-α-specific forward: 5′-TCTTCTCATTCCTGCTTGTGG-3′,

TNF-α-reverse primer: 5′-GGTCTGGGCCATAGAACTGA-3′,

IL-12-specific forward: 5′-CCTCAGTTTGGCCAGGGTC-3′,

IL-12-reverse primer: 5′-CAGGTTTCGGGACTGGCTAAG-3′,

IL-6-specific forward: 5′-GATGGATGCTACCAAACTGGAT-3′,

IL-6-reverse primer: 5′-CCAGGTAGCTATGGTACTCCAGA-3′,

IL-10-specific forward: 5′-ACCTGCTCCACTGCCTTGCT-3′,

IL-10-reverse primer: 5′-GGTTGCCAAGCCTTATCGGA-3′,

TGF-β1-specific forward: 5′-CTCCCGTGGCTTCTAGTGC-3′,

TGF-β1-reverse primer: 5′-GCCTTAGTTTGGACAGGATCTG-3′,

18S rRNA-specific forward: 5′-CGCCGCTAGAGGTGAAATTCT-3′,

18S rRNA-reverse primer: 5′-CGAACCTCCGACTTTCGTTCT-3′.

18S rRNA was used as a reference gene. All real-time PCR reagents were purchase from Bio-Rad and all reactions were run on a CFX96 Real-Time System (Bio-Rad, CA, USA). The remaining tissue was weighed and re-suspended with RIPA buffer containing 1% protease inhibitor cocktail (Sigma-Aldrich, MO, USA) for 2 hours, and then centrifuged at 17,000 × g for 20 minutes at 4 °C. Tissue supernatants were collected for luminex (Merck Millipore, MA, USA) and ELISA testing.

### Immunofluorescence

2 × 10^7^ GFP^+^ PCs were intravenously injected into the colitis mice at day7. After 1 hour, colon was made into frozen sections immediately. The PE-conjugated antibodies to F4/80 and CD5, and DAPI were used in immunofluorescence. Fluorescence expression was directly observed by DM600 confocal microscope system (DM600 CS, Leica, Germany).

### Flow cytometry

For detecting macrophage and B cells *in vivo*, tissues were minced and digested with 1 mg/ml collagenase I in 37 °C for 2 h. After erythrocyte lysis, the cells were washed with PBS and stained with the primary antibodies (BD Bioscience and BioLegend) including: anti-CD11b, anti-F4/80, anti-Ly6C, anti-B220 and anti-CD5, anti-CD19, anti-CD1d, anti-CD45, anti-MHCII, anti-CD3, and anti-CD11c. Cells were stained with primary antibodies and then analyzed with FACS Aria II (BD Biosciences, San Jose, CA, USA). The specific CD19 + CD1d + CD5 + B cells (population #1), CD19 + CD1d-CD5 + B cells (population #2), CD45 + MHCII + CD3-CD19-F4/80 + anti-inflammation macrophages (population #3) and CD45 + MHCII + CD3-CD19-F4/80-CD11c + inflammatory DCs (population #4) were sorted from DSS + PC group by BD FACS Aria II and then measured the mRNA levels of IL-10 and TGF-β1 by RT-PCR.

### Western blotting

Colon tissues were immediately frozen in liquid nitrogen and stored at −80 °C. The whole colon of mice was homogenized in lysis buffer with 1% protease inhibitor cocktail (Sigma-Aldrich, St. Louis, MO, USA). The extracts were centrifuged at 13,000 rpm at 4 °C for 20 min and then determined the protein contents by BCA protein assay. Cytosolic extracts (100 μg) were fractionated by 10% sodium dodecyl sulfate-polyacrylamide gel electrophoresis. Anti-STAT3, anti-p-STAT3, anti-Smad3, anti-p-Smad3 and anti- NF-κB were purchased from Abcam (Cambridge, MA, USA). Anti-p-NF-κB was purchased from cell signaling (Boston, MA, USA). Smad7 was purchased from R&D (R&D Systems, Minnesota, USA). Rabbit polyclonal and mouse monoclonal anti-β-actin were obtained from ZSGB-BIO (ZSGB-Biotechnology Company, Beijing, China). T cells in colitis colon were isolated by mouse CD4 + T cell enrichment kit (Stemcell Technology, CAN).

### Mice immunized with OVA

Mice were randomly divided into two groups: the OVA-sensitized group (OVA) and the OVA-sensitized and PCs-treated group (OVA + PC). Mice in each group were administered 20 μg OVA (Sigma-Aldrich) by subcutaneous injection on days 0 and 14^48^. Additionally, the OVA and OVA + PC groups were treated with saline or 1 × 10^6^ peritoneal cells, respectively. Whole blood was collected from the retro-orbital venous plexus on days 10 and 21. The amount of OVA-specific IgG in serum was determined by ELISA. Briefly, 96-well plates were coated with OVA at 10 μg/well overnight. After the plates were blocked with 5% milk (Bio-Rad), 100 μl/well of diluted serum was added and the plates were incubated at 37 °C for 2 hours. After incubation, plates were washed five times with PBST (PBS containing with 0.5% Tween-20 buffer) and the secondary antibody (goat anti-mouse IgG, diluted at 1:10000, Abcam, MA, USA) was added. Plates were incubated for 1 hour at 37 °C. After plates were again washed, 100 μl TMB (KPL, USA) was added and samples were incubated in the dark at room temperature for 20 min. The reaction was stopped by adding 100 μl/well of 2N H_2_SO_4_ and measured at 450 nm using a microplate autoreader (Multiskan Mk3 ELISA reader, Thermo).

### *In vitro* culture

Isolated PCs, peritoneal macrophages and B cells were re-suspended in RPMI-1640 at the concentration of 4 × 10^5^ cells/ml. Cells were cultured in 24-well plates at 37 °C, 5% CO_2_ for 6 hours. RPMI-1640 medium was used as control. The levels of IL-10 and TGF-β1 in the supernatants were measured using a commercial ELISA kit (eBioscience, CA, USA). BM flushing from femur and tibia bones was separated by Histopaque-1077 (Sigma-Aldrich, MO, USA). The collected lymphocytes and other mononuclear cells were further cultured for 24 hours with 20 ng/mL TGF-β-1 and/or IL-10 (PeproTech, NJ, USA)^28^,^49^. Then, the cells were collected for FACS analysis.

### Neutralizing antibody administration

Mice in DSS + PC group were further intraperitoneally injected with 50 μg mouse anti-IL-10 and/or anti-TGF-β-1, -2, -3 antibodies (MAB417 and MAB1835, R&D Systems, Minnesota, USA) on day 0. This regimen was followed by intraperitoneal injections of 100 μg antibodies on day 2, and an additional 50 μg antibody on days 4 and 6. Control groups received equivalent amounts of rat-IgG or mouse-IgG (R&D Systems), following the same protocol^50^.

### Statistical analysis

The SPSS statistical software was used to analysis. Results were presented as means ± SEM unless otherwise stated. Statistical significance was determined by Student’s *t-*test and two-way analysis of variance (ANOVA) or the Wilcoxon Mann–Whitney test for non-parametrically distributed samples. P < 0.05 was considered to represent statistically significant differences.

## Additional Information

**How to cite this article**: Liu, T. *et al.* Treatment of dextran sodium sulfate-induced experimental colitis by adoptive transfer of peritoneal cells. *Sci. Rep.* **5**, 16760; doi: 10.1038/srep16760 (2015).
